# Clinical classification of tissue perfusion based on the central venous oxygen saturation and the peripheral perfusion index

**DOI:** 10.1186/s13054-015-1057-8

**Published:** 2015-09-14

**Authors:** Huaiwu He, Yun Long, Dawei Liu, Xiaoting Wang, Xiang Zhou

**Affiliations:** Department of Critical Care Medicine, Peking Union Medical College Hospital, Peking Union Medical College, Chinese Academy of Medical Science, 1 shuaifuyuan, Dongcheng District 100730 Beijing, China

## Abstract

**Introduction:**

We investigated whether combining the peripheral perfusion index (PI) and central venous oxygen saturation(ScvO_2_) would identify subsets of patients for assessing the tissue perfusion and predicting outcome during the resuscitation in critically ill patients.

**Methods:**

A total of 202 patients with central venous catheters for resuscitation were enrolled in this prospective observational study. The arterial, central venous blood gas and the PI were measured simultaneously at the enrollment (T0) and 8 h (T8) after early resuscitation. Based on the distribution of the PI in healthy population, a cutoff of PI ≥1.4 was defined as a normal PI. Moreover, the critical value of PI was defined as the best cutoff value related to the mortality in the study population. The PI impairment stratification is defined as follows: a normal PI(≥1.4), mild PI impairment (critical value < PI < 1.4) and critical PI impairment (PI ≤ critical value).

**Results:**

The PI at T8 was with the greatest AUC for prediction the 30-day mortality and PI is an independent risk factor for 30-day mortality. Moreover, a cutoff of PI < 0.6 is related to poor outcomes following resuscitation. So, based on cutoffs of ScvO_2_ (70 %) and critical PI (0.6) at T8, we assigned the patients to four categories: group 1 (PI ≤ 0.6 on ScvO_2_ < 70 %), group 2 (PI ≤ 0.6 on ScvO_2_ ≥ 70 %), group 3 (PI > 0.6 on ScvO_2_ < 70 %), and group 4 (PI > 0.6 on ScvO_2_ ≥ 70 %). The combination of low ScvO_2_(<70 %) and PI(≤0.6) was associated with the lowest survival rates at 30 days [log rank (Mantel–Cox) = 87.518, p < 0.0001]. The sub-group patients who had high ScvO_2_(>80 %) at T8 were with low mortality and high PI. Moreover, the normal PI (≥1.4) did not show a better outcome than mild impaired PI (0.6-1.4) patients who had a normalized ScvO_2_(>70 %) after resuscitation. The PI was correlated with the lactate, P(v-a)CO_2_, and ScvO_2_ in all the measurements (n = 404). These relationships are strengthened with abnormal PI (PI < 1.4) but not with normal PI (PI ≥ 1.4).

**Conclusion:**

Complementing ScvO_2_ assessment with PI can better identify endpoints of resuscitation and adverse outcomes. Pursuing a normalized PI (≥1.4) may not result in better outcomes for a mild impaired PI after ScvO_2_ is normalized.

**Electronic supplementary material:**

The online version of this article (doi:10.1186/s13054-015-1057-8) contains supplementary material, which is available to authorized users.

## Introduction

Global oxygen metabolism perfusion measurements that are derived from blood gas analysis and peripheral circulation perfusion assessment are frequently used practical methods to determine tissue perfusion. Central venous oxygen saturation (ScvO_2_) has been widely accepted as an indicator to reflect the balance between global oxygen demand and oxygen supply, and a cutoff of 70 % for ScvO_2_ has become an endpoint of early resuscitation [1]. However, recent negative results from early goal-directed therapeutic studies have questioned whether ScvO_2_ equal to or greater than 70 % should be the goal of early resuscitation [2, 3]. Conversely, pursuing normalization of ScvO_2_ may result in adverse effects. It is challenging to determine the most appropriate criteria to stop resuscitation opportunely so as to avoid the risk of over-resuscitation. Moore et al. suggested that the concept of early goal-directed therapy should be revised, and the inclusion of the restoration of peripheral circulation perfusion in the early resuscitation would be meaningful [4].

With the development of medical techniques, quantitative assessment of peripheral tissue perfusion has become prevalent in clinical practice [5–7]. Studies have shown that persistent impairment in peripheral circulation perfusion is related to a poor outcome in critically ill patients [8, 9]. Inconsistencies in systemic oxygen metabolism variables and peripheral circulation perfusion have been investigated quite intensively in recent years [10]. The change in finger peripheral perfusion index (PI) results from the blood volume pulsations, the dispensability of the vascular wall and the intravascular pulse pressure [11]. It has been suggested as a reliable and early indicator of the success of regional block, and is known to increase due to the effect of autonomic blockade during spinal anesthesia [12]. The PI has been shown to reflect changes in peripheral circulation perfusion and central hypovolemia, which is derived from the photoelectric plethysmographic pulse oximetry signal [13, 14]. Compared with the distribution of the PI in a healthy adult population, a cutoff of PI <1.4 is used as a very sensitive point for identifying abnormal peripheral perfusion associated with vasoconstriction in critically ill patients [14]. Some studies have used abnormal PI <1.4 as a potential trigger to start treatment. However, the question as to whether an abnormal PI value <1.4 requires total correction is undetermined.

The sacrifice of peripheral tissue is a self-protecting mechanism for the vital organs during shock. It is assumed that peripheral tissue is the first tissue bed to be sacrificed during shock and the last to be re-perfused in resuscitation. Therefore, we speculated that there would be a critical PI value related to mortality, which works as the safe limit. Thus, we hypothesized that the combination of ScvO_2_ (to determine whether the oxygen supply is sufficient or insufficient) and PI (to determine the severity of peripheral perfusion: normal, mild impairment, critical impairment) would provide additional information for predicting outcomes and endpoints of resuscitation.

The aims of the study were the following: 1) to define a critical value of PI related to mortality after resuscitation in critically ill patients; 2) to define a prognostic value of the preset clinical classification according to the normal ScvO_2_ (70 %) and critical value of PI after resuscitation; and 3) to define a prognostic value based on the stratification of the severity of PI after ScvO_2_ (≥70 %) normalization.

## Methods

### Patients

The Institutional Research and Ethics Committee of the Peking Union Medical College Hospital approved this study for human subjects. Written informed consent was obtained from all patients or next of kin before data were included in the study. When the research team was available, adult patients within 24 h after the onset of suspected clinical tissue hypoperfusion who were sequentially admitted to the Department of Critical Care Medicine and required central venous catheters for resuscitation were eligible for the study. The placement of a central venous catheter for resuscitation was determined by the attending physician according to the clinical situation. Patients were excluded from the study if they were pregnant, aged <18 years, were not expected to survive, were brain dead, or had made a decision to withhold or withdraw treatment. The inclusion time (T0) and the study enrollment were considered to begin the moment the central venous pressure monitoring began after ICU admission.

The tissue hypoperfusion diagnostic criteria were the following: 1) SBP ≤90 mmHg (or a decrease in SBP ≥20 % from baseline); 2) urinary output <0.5 ml/kg/min for more than 2 h; 3) increase in heart rate (HR) ≥10 % from baseline; 4) presence of skin mottling; and 5) hyperlactatemia (>2 mmol/L). If one or more of these criteria were met, clinical tissue hypoperfusion was diagnosed. The physician would make a decision to begin resuscitation based on the clinical situation. Septic shock was defined as severe sepsis with sepsis-induced hypotension persisting despite adequate fluid resuscitation and requiring the administration of vasopressors [15].

All the patients received a local hemodynamic support algorithm for critically ill patients. The early goals of hemodynamic support are the following: central venous pressure of 8–12 mmHg; mean arterial pressure >65 mmHg; urine output >0.5 ml/kg of body weight (except in the patients with acute renal failure); and ScvO_2_ of 70 % or more with veno-arterial CO_2_ tension difference (P(v-a)CO_2_) of 6 mmHg or less. The intensivists were blinded to the results of the PI.

### Measurements

Information collected at enrollment included demographic characteristics, such as age, sex, acute physiology and chronic health evaluation II score (APACHE II) [16]; global hemodynamic, arterial and central venous blood gas analysis and PI were obtained simultaneously at T0 and T8 (approximately 8 h (+/−1 h) after early resuscitation was deemed to meet the requirements).

A central venous catheter was inserted via the jugular or the subclavian vein, and the position of the tip of the venous catheter was in the upper part of the right atrium verified by chest radiograph. Blood gas samples were taken anaerobically in 3-ml heparinized syringes and analyzed on a bedside blood gas machine (GEM Premier 3000, model 5700, Lexington, MA, USA or ABL90, Radiometer, Copenhagen, Denmark), and the same blood gas machine was used to measure both the arterial and central venous blood gas. PI was measured in the finger by using the IntelliVue MP70 monitor (Philips Medical Systems, Boblingen, Germany). The MP70 system calculates the PI as the ratio between the pulsatile component and the non-pulsatile component of the light reaching the light-sensitive cell of the pulse oximetry probe. The ambient temperature of the room was consistent at approximately 23 to 25 °C (climate controlled). Survival was defined as being alive for 30 days after enrollment.

### Study definitions

- Normal ScvO_2_ was defined as ≥70 %, and normal PI was defined as ≥1.4
- The critical value of PI was derived from the best cutoff value related to the mortality in the study population
- The patients were divided into four categories based on the normal ScvO_2_ and critical PI value
- PI impairment stratification was as follows: normal PI (≥1.4), mild PI impairment (critical value < PI <1.4) and critical PI impairment (PI ≤ critical value, which was defined as mentioned above).

### Statistical analysis

Descriptive analysis was performed. All data are expressed as the mean ± standard deviation or median (25th–75th percentiles) unless otherwise specified. For the continuous variables, data were analyzed using the *t* test, analysis of variance (ANOVA), Mann–Whitney test, or Kruskal–Wallis test depending on their distribution and number of variables. Comparisons of two continuous variables were performed using linear regression. Variables were introduced into a multivariable binary logistic regression model if significantly associated with mortality at day 30, using the univariate analysis when the *p* value was <0.2. General demographics, hemodynamics, norepinephrine (NE) dose, PI and blood gas parameters at T0 and T8 were used in the model. Discrimination of values was performed using receiver operating characteristic (ROC) analysis. Survival curves up to day 30 were estimated using the Kaplan–Meier method, and the log rank (Mantel–Cox) test was used to estimate differences among the predefined groups. Repeated measurements were analyzed using analysis of variance or analysis of variance on ranks. The chi-squared (*χ*^2^) test (or Fisher’s exact test when appropriate) was used to compare discrete variables. All comparisons were two-tailed, and *p* <0.05 was required to exclude the null hypothesis. The statistical analysis was performed using the SPSS 13.0 software package (SPSS Inc., Chicago, IL, USA). The area under the ROC curve was compared using the Hanley − McNeil test [17].

## Results

**Fig. 1:**
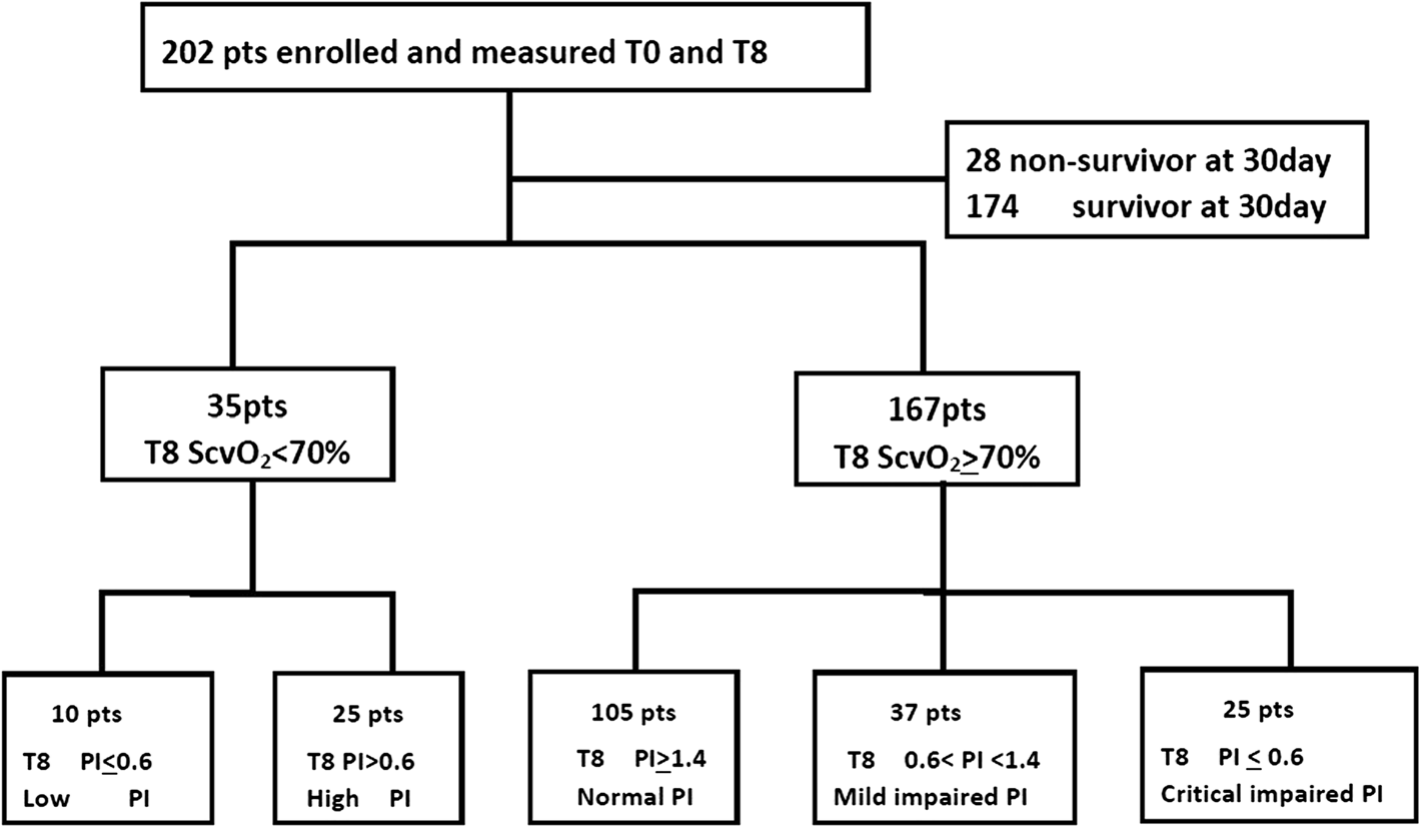
Flow diagram showing the 202 patients stratified according to central venous oxygen saturation (*ScvO*_*2*_) and peripheral perfusion index (*PI*) at 8 h after resuscitation (*T8*). *pts* patients, *T0* baseline

During the study period (from November 2013 to February 2014), a total of 344 patients were admitted to our department, and 202 critically ill patients were enrolled in this study. Therefore, a total of 404 measurements were obtained at T0 and T8 in the 202 patients. The mortality at day 30 in this cohort was 28/202 (14 %), and length of ICU stay was 6 ± 10 days. The demographics and clinical characteristics of all the patients are shown in Table 1. The distribution of all PI measurements was skewed, and values ranged from 0 to 8 with a median of 1.33 and an interquartile range of 2. The flow diagram in Fig. 1 shows the 202 patients according to ScvO_2_ and PI at T8 after resuscitation. The median time between ICU admission and enrollment (T0) was 1 h.

**Table 1:** Characteristics of the patients

Number of patients	202
Age, years	57 ± 18
Sex, n, female/male	104/98
Baseline PaO_2_, mmHg	142 ± 72
Baseline Hb, g/dl	11 ± 3
Admission ward	
Medical	15
Surgical	146
Emergency	42
Admission category	
Sepsis	84
Postoperative with high risk	96
Other	22
Baseline APACHE II score	13 (9–20)
Baseline SOFA score	8 (5–10)
Baseline mechanical ventilation, % (n/total)	85 % (171/202)
Baseline NE, % (n/total)	37 % (75/202)
Baseline NE dose, ug/kg/min	0.11 ± 0.23
NE, % (n/total) at T8	48 % (96/202)
NE dose at T8, ug/kg/min	0.22 ± 0.63
ICU LOS, days	6 ± 10
Survivor/non-survivor at 30 days n/total (%)	28/202 (14 %)

Results are presented as mean ± SD or median (IQR) unless stated otherwise

*Hb* hemoglobin concentration, *PaO*_*2*_ arterial oxygen tension, *APACHE* acute physiology and chronic health evaluation, *SOFA* sequential organ failure assessment score, *LOS* length of stay, *NE* norepinephrine

### Prediction of mortality at day 30

**Fig. 2:**
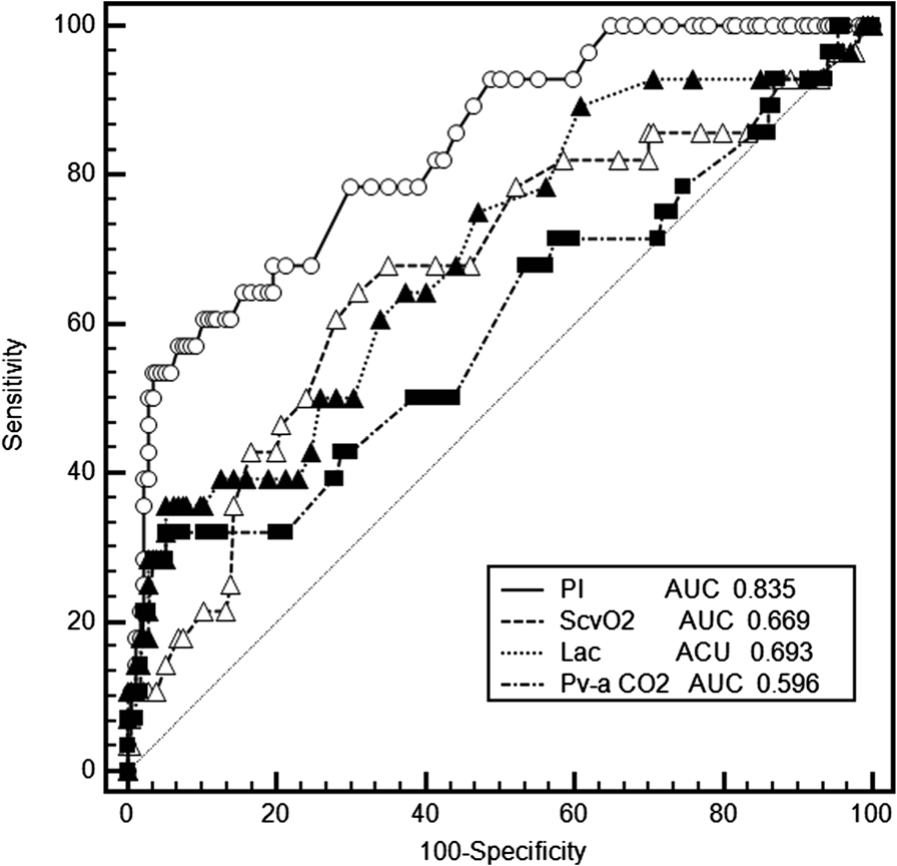
Receiver operating characteristic curves comparing the ability of peripheral perfusion index (*PI*), lactate (*Lac*), difference between central venous and arterial PCO_2_ (*Pv-a CO*_*2*_) and central venous O_2_ saturation (*ScvO*_*2*_) to discriminate mortality at day 30 in all the critically ill patients. *AUC* area under the curve

There were no differences in MAP, CVP, age, ScvO_2_, PI, lactate, or difference between central venous and arterial PCO_2_ (P(v-a)CO_2_) at baseline (T0) between the survivors and non-survivors. In addition, statistically significant variables between the survivors and the non-survivors included ScvO_2_, PI and lactate at T8 after early resuscitation. The AUC of the related variables used to predict 30-day mortality are shown in Table 2. The ROC curves of PI, lactate, ScvO_2_ and P(v-a)CO_2_ are shown in Fig. 2. The PI at T8 was with the greatest AUC for prediction of 30-day mortality and was significantly better than ScvO_2_, P(v-a)CO_2_ and lactate. A PI threshold of 0.6 was associated with a sensitivity of 60.71 % and a specificity of 89.66 % for predicting 30-day mortality.

**Table 2:** Receiver operating characteristic (ROC) curve analysis of the different variables at 8 h (T8) to predict mortality at 30 days

Variable	ROC area	95 % CI	Cutoff value	Sensitivity	Specificity
Lactate, mmol/L	0.693*	0.625, 0.756	3.6	35.71	94.83
ScvO_2_, %	0.669*	0.599, 0.733	70	42.86	83.33
P(v-a) CO_2_	0.596*	0.525, 0.664	6	42.86	70.11
PI	0.835	0.777, 0.884	0.6	60.71	89.66

*P(v-a)CO*_*2*_, difference between central venous and arterial PCO_2_ (mmHg), *PI* peripheral perfusion index measured by pulse oximetry, *ScvO*_*2*_ central venous O_2_ saturation

*P <0.05 for comparison of PI vs ScvO_2_, P(v-a) CO_2_, and lactate

Furthermore, there were 35 patients with PI ≤0.6 and 167 patients with PI >0.6 at T8 after resuscitation. There were no differences in temperature, ScvO_2_, MAP or CVP between patients with PI ≤0.6 and patients with PI >0.6. Patients with PI ≤0.6 had higher lactate and P(v-a)CO_2_ at T8 after resuscitation. The related data are shown in Additional file 1.

### Clinical classification based on ScvO_2_ (70 %) and critical PI (0.6) at T8

**Fig. 3:**
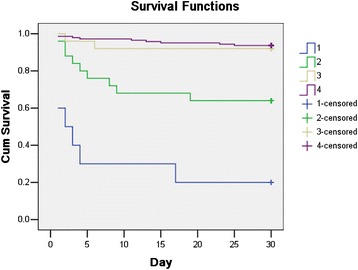
Survival probabilities up to day 30 according to central venous O_2_ saturation (ScvO_2_) and peripheral perfusion index (PI) after 8 h of resuscitation. Log rank (Mantel–Cox) = 87.518, *p* <0.0001. Group 1 (PI ≤0.6 with ScvO_2_ < 70 %); group 2 (PI ≤0.6 with ScvO_2_ ≥ 70 %); group 3 (PI >0.6 with ScvO_2_ < 70 %); and group 4 (PI >0.6 with ScvO_2_ ≥ 70 %). *Cum survival* cumulative survival

**Fig. 4:**
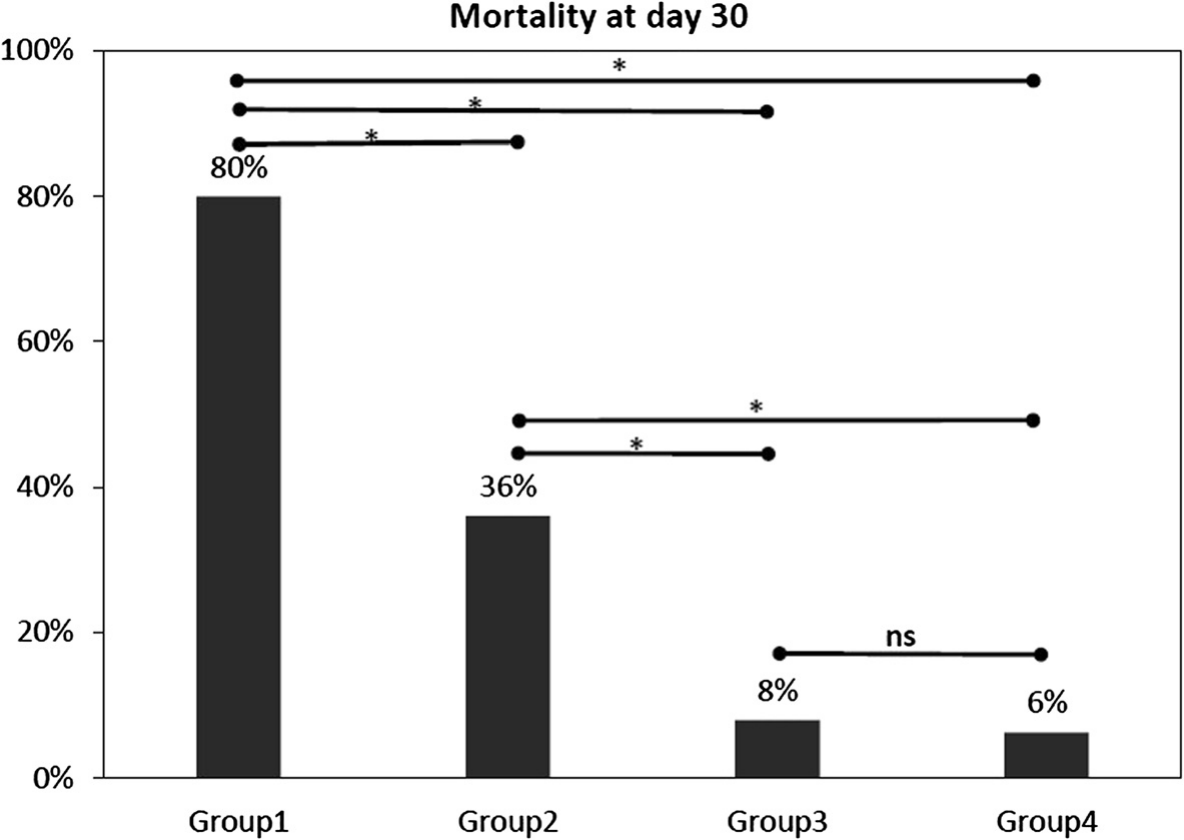
Mortality at day 30 for predefined groups based on PI(0.6) and ScvO_2_(70 %) at T8 after resuscitation. *p < 0.05. ns, no significance. group 1 (PI ≤ 0.6 with ScvO_2_ < 70 %); group 2 (PI ≤ 0.6 with ScvO_2_ ≥ 70 %); group 3 (PI > 0.6 with ScvO_2_ < 70 %); and group 4 (PI > 0.6 with ScvO_2_ ≥ 70 %)

According to the preset principle, the patients were assigned to four categories: group 1 (PI ≤0.6 on ScvO_2_ < 70 %), group 2 (PI ≤0.6 on ScvO_2_ ≥ 70 %), group 3 (PI >0.6 on ScvO_2_ < 70 %), and group 4 (PI >0.6 on ScvO_2_ ≥ 70 %). Group 1 (low PI and low ScvO_2_) had the lowest survival rate at day 30 (log rank (Mantel–Cox) = 87.518, *p* <0.0001) (Fig. 3), which was with the highest lactate level at T8 after resuscitation. The mortality at day 30 of groups are shown in the Fig. 4, and group 2 (low PI and normal ScvO_2_) had the second highest mortality. No differences in initial lactate levels were observed between the four groups. Moreover, there was no difference in lactate, PI or 30-day mortality between group 3 (normal PI and low ScvO_2_) and group 4 (normal PI and normal ScvO_2_). The clinical characteristics of the different groups are shown in Additional file 2.

In addition, patients were divided into three groups according to the ScvO_2_ value at T8 after resuscitation (low ScvO_2_ was defined as ScvO_2_ < 70 %, normal ScvO_2_ was defined as 70 % ≤ ScvO_2_ ≤ 80 %, high ScvO_2_ was defined as ScvO_2_ > 80 %) (Table 3). The patients who had low ScvO_2_ had a low PI, a high P(v-a)CO_2_ and high mortality, and vice versa. Moreover, the patients with a low PI (≤0.6) had low lactate clearance in sub-analysis of the high ScvO_2_ group after resuscitation.

**Table 3:** Related variables and outcome of different groups according to ScvO_2_ level at 8 h (T8) after resuscitation

	Low ScvO_2_ < 70 %	Normal 70 ≤ ScvO_2_ ≤ 80	High ScvO_2_ > 80 %
	*n* = 35	*n* = 90	*n* = 77
P(v-a) CO_2_ at T8	8 ± 4*^,^**	6 ± 3**	5 ± 4
Lactate at T8, mmol/L	2.9 ± 3.8	1.9 ± 1.8	1.7 ± 1.7
8-h LC, %	13 (−3 to 50)	26 (1 to 50)	40 (1 to 57)
PI at T8	1.9 ± 1.7**	2.0 ± 1.3**	2.5 ± 1.6
Patients, n/total (%), PI ≤0.6 at T8	10/35 (29 %)	15/90 (17 %)	10/77 (13 %)
Mortality at day 30, n/total (%)	10/35 (29 %)	13/90 (14 %)	5/77 (6 %)

Results are presented as mean ± SD or median (IQR) unless stated otherwise

*ScvO*_*2*_ central venous oxygen saturation (%), *P(v-a) CO*_*2*_ veno-arterial CO_2_ tension difference (mmHg), *PI* peripheral perfusion index, *LC* lactate clearance

*P <0.05 for comparison to normal

**p <0.05 for comparison to high

### Multivariable logistic regression analysis

Multivariable logistic regression analysis at T8 demonstrated that PI was an independent predictor of mortality at day 30 (relative risk (RR) 2.439; 95 % CI 1.296-4.589, P =0.006). When analysis was performed using the same variables at T0, PI was not related to higher mortality at day 30 (Table 4).

**Table 4:** Multivariate logistic regression for predictors of 30-day mortality at baseline (T0) and 8 h (T8)

Variables	T0			T8		
	RR	95 % CI	*P* value	RR	95 % CI	*P* value
PI	1.055	0.685, 1.691	0.825	2.439	1.296, 4.589	0.006
Lactate	1.118	0.868, 1.439	0.388	1.228	0.886, 1.702	0.218
ScvO_2_,%	0.990	0.936, 1.046	0.710	1.080	1.007, 1.158	0.032
P(v-a)CO_2_, mmHg	0.981	0.866, 1.112	0.767	0.975	0.828, 1.148	0.763
HR	0.936	0.909, 0.965	0.000	0.964	0.939, 0.991	0.008
CVP	0.922	0.792, 1.074	0.298	1.059	0.890, 1.259	0.519
MAP	1.016	0. 983, 1.051	0.333	1.017	0.975, 1.060	0.434
Age, years	0.968	0.935, 1.002	0.068	0.998	0.967, 1.030	0.899
Gender	0.496	0.172, 1.434	0.196	0.396	0.128, 1.224	0.108
NE dose, ug/kg/min	0.146	0.022, 0.969	0.046	0.103	0.015, 0.692	0.019

*CVP* central venous pressure, *MAP* mean arterial pressure, *HR* heart rate, *ScvO2* central venous oxygen saturation, *P(v-a) CO2* difference in venoarterial CO2 tension, *NE* norepinephrine, *PI* peripheral perfusion index

### Relationship between PI and blood gas metabolic parameters

The PI was significantly correlated with lactate (*r* = −0.261, *p* <0.0001), P(v-a)CO_2_ (*r* = −0.152, *p* <0.0001), and ScvO_2_ (*r* = 0.190, *p* <0.0001) in all of the measurements (n = 404). In the sub-measurements of PI <1.4 (n = 204), these relationships seem to be slightly strengthened. However, in the sub-measurements of PI ≥1.4 (n = 200), these relationships were lacking (shown in Additional file 3).

### PI impairment stratification after normalized ScvO_2_ (≥70 %) at T8

After resuscitation, there were 167 patients with normalized ScvO_2_ at T8. There were significant differences between survivors and non-survivors at day 30 in the time course of Pv-aCO_2_, lactate, and PI from T0 to T8 during the resuscitation (repeated measurements ANOVA, *p* <0.05) but not ScvO_2_.

Stratification by the severity of PI impairment was defined as follows: normal PI (≥1.4), mild PI impairment (0.6 < PI <1.4), and critical PI impairment (≤0.6). According to the classification of the severity of PI impairment at T8, the clinical characteristics and outcome of patients with different classifications of severity of impairment of PI are shown in Table 5. The patients classified as having critical PI impairment had higher lactate and APACHE II scores and lower 8-h lactate clearance. However, there was no difference between the patients with normal or mild PI impairment in lactate at T8, 8-h lactate clearance or the sequential organ failure assessment (SOFA) or APACHE II scores.

**Table 5:** Related variables and outcome in different groups according to stratification by severity of impairment of PI at T8 after normalization of ScvO_2_ (≥70 %)

		Critical	Mild	Normal
		PI ≤0.6	0.6 < PI <1.4	PI ≥1.4
		*n* = 25	*n* = 37	*n* = 105
T0	CVP, mmHg	9 ± 4	9 ± 3	10 ± 3
	MAP	94 ± 20	90 ± 16	91 ± 17
	ScvO_2_, %	71 ± 13	74 ± 11	77 ± 9
	P(v-a) CO_2_, mmHg	8 ± 4**	6 ± 4	6 ± 3
	Lactate, mmol/L	3.2 ± 3.1	2.6 ± 1.8	2.8 ± 2.2
	PI	0.6 ± 0.6*^,^**	0.9 ± 0.6**	1.8 ± 1.3
T8	CVP, mmHg	9 ± 4	9 ± 3	8 ± 3
	MAP	87 ± 15	88 ± 10	86 ± 13
	ScvO_2_, %	80 ± 4	79 ± 6	80 ± 6
	P(v-a) CO_2_, mmHg	7 ± 5	5 ± 3	5 ± 3
	Lactate, mmol/L	2.9 ± 3.2**	1.8 ± 1.4	1.5 ± 1.2
	PI	0.4 ± 0.2*^,^**	1 ± 0.2**	3.1 ± 1.2
Base APACHE II		20 (12 to 29)*	11 (8 to 21)	12 (7 to 16)
Base SOFA		9 (7 to 10)**	7 (5 to 11)	7 (4 to 9)
Mortality at day 30, n/total (%)		9/25 (35 %)*^,^**	4/37 (11 %)	5/105 (5 %)
8-h LC, %		14 (−45 to 38)**	25 (−2.5 to 47)	43 (0 to 60)

Results are presented as mean ± SD or median (IQR) unless stated otherwise

*APACHE* acute physiology and chronic health evaluation, *SOFA* sequential organ failure assessment score, *CVP* central venous pressure, *MAP* mean arterial pressure, *ScvO*_*2*_ central venous oxygen saturation, *P(v-a) CO*_*2*_ veno-arterial CO_2_ tension difference, *PI* peripheral perfusion index, *LC* lactate clearance

*P <0.05 for comparison to mild-moderate

**p <0.05 compared to normal

**Fig. 5:**
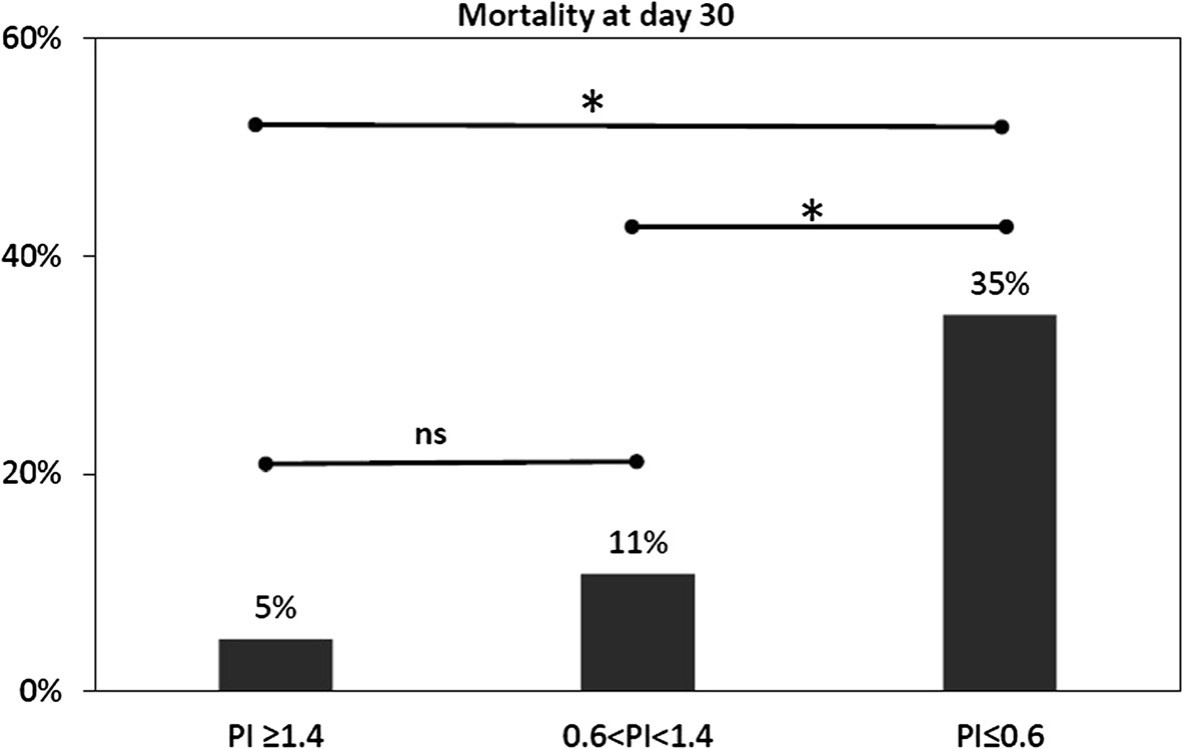
Mortality at day 30 according to stratification by severity of impairment of peripheral perfusion index (*PI*) after normalization of central venous oxygen saturation (ScvO_2_) (≥70 %) at 8 h (T8). *P <0.05; *ns* not significant. PI ≥1.4 (normal PI, n = 105); 0.6 < PI <1.4 (PI mild impairment n = 37); PI ≤0.6 (PI critical impairment, n = 25)

Compared with patients with normal or mild PI impairment, patients with critical PI impairment had the greatest 30-day mortality (30-day mortality: normal PI vs critical PI, 5/105 vs 9/25, *χ*^2^ = 19.459, *p* <0.0001; mild PI vs critical PI, 9/25 vs 4/37, *χ*^2^ = 5.284, *p* = 0.029). However, there was no difference in 30-day mortality between the patients with normal or mild PI impairment (30-day mortality: normal PI vs mild PI, 5/105 vs 4/37, *χ*^2^ = 1.686, *p* = 0.24) (Fig. 5).

## Discussion

To the best of our knowledge, this prospective, observational study is the largest reported to investigate the PI prognostic value in critically ill patients. We showed the following: (a) PI is an independent risk factor for 30-day mortality, and a cutoff of PI <0.6 is related to poor outcome following resuscitation; (b) after normalizing ScvO_2_ (≥70 %), patients with critical PI impairment (≤0.6) had the worst outcome, but there was no difference in outcome between patients with normal PI (≥1.4) and mild PI impairment (0.6 < PI <1.4); and (c) the PI correlated with lactate, P(v-a)CO_2_, and ScvO_2_. In addition, these relationships seem to be strengthened with abnormal PI (PI <1.4) but not with normal PI (PI ≥1.4).

It is well-known that persistent abnormalities in peripheral perfusion are associated with a poor outcome and multiple organ dysfunction, and regional tissue perfusion variables are better than global variables for predicting ICU mortality [18–24]. We also found the patients with poor PI (≤0.6) had higher lactate and worse outcomes. Surprisingly, we found that the PI had the greatest AUC for prediction of 30-day mortality after resuscitation, which was significantly better than lactate in the mixed critically ill patients. We inferred that in the heterogeneous patients there might be impairment of the ability of lactate to predict the mortality, but the PI still work. Additionally, we showed the PI at T8 is related to the outcome, but not PI at T0. This was consistent with the work of Poeze and colleagues. These researchers also reported that gastric tonometry variables were predictive of outcome at the normalization of global hemodynamics after resuscition, but not at the initiation of resuscitation [23].

### The classification of severity of PI impairment

As we known, the classification of the severity of PI impairment was first defined in critically ill patients. The sacrifice of peripheral perfusion is a self-protection mechanism in the critical condition, so the impairment of PI might be acceptable to some extent. The peripheral perfusion circulation is estimated in a binary fashion (good or poor), which might be insufficient in clinical practice.

To exclude the contribution of insufficient global oxygen delivery (DO_2_) to low peripheral perfusion, we analyzed the classification of severity of PI impairment in patients with normalized ScvO_2_ (≥70 %) after resuscitation. We found that patients with a normal PI (>1.4) did not have a better outcome than the patients with mild PI impairment (0.6 − 1.4) after resuscitation. Therefore, we speculated that pursuing total normalization of peripheral perfusion might induce over-resuscitation but might not improve the outcome after normal ScvO_2_ is achieved. In other words, pursuing a better physiological status is not necessarily associated with a better outcome. We suggest that mild PI (0.6 − 1.4) impairment might be “permissive impaired peripheral perfusion” and does not call for immediate and aggressive resuscitation. There is a wide variability in the course of individual recovery after optimization of DO_2_ in clinical practice, and recently the indication for stopping resuscitation has been emphasized [25]. Focus has been on the application of a microvascular circulation perfusion indicator to identify the endpoint during the resuscitation [26]. However, the greatest challenge of goal-directed microcirculation or local peripheral perfusion therapy is the definition of critical value. Animal and human studies have showed that fluid therapy guided by local perfusion parameters could reduce fluid administration and avoid overtreatment, but not affect the outcome [27, 28]. The critical value of PI defined in this paper needs further interventional studies using peripheral perfusion as a target for resuscitation.

### The relevance of the combination of ScvO_2_ and PI

The concept of the relationship between DO_2_/VO_2_ was a milestone in shock resuscitation, and determining the dependence of DO_2_/VO_2_ is the core idea in managing shock resuscitation [29–31]. The ScvO_2_ remains a simple indicator of the global balance of DO_2_/VO_2_, although this idea has been strongly challenged by others [2, 3, 32, 33]. In our study, the percentage of patients with low ScvO_2_ was small at T0, and it decreased significantly after early resuscitation at T8 (T0 vs T8, 30 % vs 9 %). Beest et al. also reported a relatively small incidence of low ScvO_2_ in critically ill patients on admission to the intensive care unit [34]. The poor outcomes of patients with low ScvO_2_ have largely been reported in various conditions. Therefore, it is not surprising that persistently poor PI combined with a low ScvO_2_ has been associated with the worst outcomes. Our study supported the pursuit of normal ScvO_2_ as the first goal in the case of persistently poor peripheral perfusion (PI ≤0.6) after resuscitation.

Recently some studies have shown that high ScvO_2_ is related to poor outcome [35–37]. However, our study did not find that high ScvO_2_ is related to high mortality after resuscitation in critically ill patients. We thought the interpretation of high ScvO_2_ should be careful in clinical practice. There are many factors that might contribute to the high ScvO_2_, and the confounders of high ScvO_2_ need to be better controlled in future studies [38]. The present study was not powered to explore the prognostic value of ScvO_2_ used as a marker of microvascular shunting or oxygen extraction dysfunction. Interestingly, we found a low PI was related to low lactate clearance in the patients with high ScvO_2_ after resuscitation. The PI still worked in the patients with high ScvO_2_. Therefore, the assessment of PI might be helpful to identify mitochondrial and microcirculatory distress syndrome in the patients with high ScvO_2_.

### The relationship between PI and metabolic variables

Our study showed that the PI was significantly positively correlated with ScvO_2_ and negatively correlated with lactate and P(v-a)CO_2_ in all the 404 measurements. The ScvO_2_, P(v-a)CO2, and lactate reflect the global DO_2_, the global blood flow and anaerobic metabolism. Conversely, the PI reflects the local peripheral circulation perfusion. Because the data were obtained in mixed critically ill patients, the correlation between PI and global metabolic variables could be interpreted as consistency between global and local variables caused by pooling the data.

Intriguingly, the lactate levels correlated strongly with a lower PI (PI <1.4) in the subset measurements. However, the relationship was lacking for normal PI (PI ≥1.4). While the presentation of normal PI might suggest sufficient perfusion, hyperlactatemia may not result from tissue hypoperfusion. Therefore, the relationship between lactate and PI was weak for normal PI (PI ≥1.4). The interpretation of hyperlactatemia in critically ill patients is complex, and factors other than hypoperfusion may be involved [39]. We inferred that peripheral circulation monitoring could provide useful information to determine the causes of hyperlactatemia (perfusion-related or non-perfusion-related hyperlactatemia) in critically ill patients. This hypothesis needs further research for clarification.

### Limitations

Several limitations should be acknowledged. First, the study period may be considered to be not long enough to evaluate other relevant clinical outcomes, and the selected time points are relatively arbitrary. Second, some may argue that the patient population was heterogeneous, which may confound this study. However, all the enrolled patients received a relative standard resuscitation treatment because the new onset of suspected tissue hypoperfusion occurred on the first day the patient was admitted to the ICU. Therefore, the data were reasonable and comparable. Third, the definition of tissue hypoperfusion might be limited and arbitrary. However, the definition of tissue hypoperfusion was commonly used in clinical practice in our study, which would always trigger medical intervention. Therefore, the standard of hypoperfusion could reflect a real clinical condition. Fourth, some may argue that septic shock might be associated with peripheral vasodilation and local hyperperfusion, which is known as “warm shock”. However, it is difficult to use PI to define tissue hyper-perfusion (lack of comparison standard), and this has not been investigated. Moreover, the roll of peripheral vasodilation and vasoconstriction in sepsis is controversial [40]. As Gilbert first noted that ‘dilation in one (vascular) bed might be accompanied by constriction elsewhere’ in 1960 [41], it is well-known that the prominent feature of sepsis is a heterogeneous distribution of blood flow. Therefore, “local tissue hyper-perfusion” might be tissue hypoperfusion but not real hyperperfusion. Our previous study showed that peripheral vasoconstriction can be a hallmark of early septic shock [9, 10]. Furthermore, there may be a correlation between peripheral vasoconstriction and unfavorable outcome. How to restore the peripheral perfusion after optimized global hemodynamic is controversial. Recently, studies showed nitroglycerin could improve the PI but not improve the outcome [42, 43]. Fifth, we acknowledge our failure to combine measurements with other peripheral tissue perfusion indicators and clinical assessment (capillary refill time/skin temperature/mottling score) in this study. There are many noninvasive techniques available to monitor peripheral circulation, and it is not yet clear which one is the best to reflect a better outcome. Studies also suggest that PI should always be used combined with clinical assessment, and there are a many extra factors that might impact the PI value [44, 45]. To reduce uncertainty around these extra factors, we strictly controlled the conditions (keeping a constant ambient temperature and obtaining the PI value without movement, suction or other extra stimulation). So, this limitation did not seem to affect the conclusion. The variation in PI response to the extra factors needs to be defined further in future research.

## Conclusion

PI is an independent risk factor for 30-day mortality following resuscitation. Successfully normalized PI (≥1.4) during treatment might be an indication to stop the resuscitation, whereas pursuing normalized PI (≥1.4) might not result in better outcomes compared with mild-to-moderate PI impairment after a normalized ScvO_2_. Complementing ScvO_2_ assessment with PI during resuscitation can better identify the endpoint of resuscitation and patients at higher risk of adverse outcomes.

## Key messages

- PI is an independent risk factor for 30-day mortality, and a cutoff of PI <0.6 is related to poor outcome following resuscitation
- Pursuing a normalized PI (≥1.4) might not result in better outcomes for mild PI impairment after normalized of ScvO_2_
- The PI was correlated with lactate, P(v-a)CO_2_, and ScvO_2_ in all of the measurements (n = 404). These relationships seem to be strengthened with abnormal PI (PI <1.4) but not with normal PI (PI ≥1.4).

## Additional files

Additional file 1:
**Results: difference between the patients with peripheral perfusion index (PI) ≤0.6 and with PI >0.6 at 8 h (T8) after resuscitation.** (DOCX 19 kb) Additional file 2:
**Results: related variables of each subset of patients based on normalized central venous oxygen saturation (ScvO**_**2**_ **) (70 %) and critical peripheral perfusion index (PI) (0.6) at 8 h (T8).** (DOCX 20 kb) Additional file 3:
**Results: relationship between peripheral perfusion index (PI) and blood gas metabolic variables on repeated measurements during resuscitation.** (DOCX 18 kb)

## Abbreviations

ANOVA: analysis of variance
APACHE II: acute physiology and chronic health evaluation II score
AUC: area under the curve
CVP: central venous pressure
DO_2_: oxygen delivery
HR: heart rate
MAP: mean arterial pressure
MV: mechanical ventilation
NE: norepinephrine (μg/kg.min)
PaO_2_: arterial oxygen tension
P(v-a)CO_2_: veno-arterial CO_2_ tension difference
PI: peripheral perfusion index
ROC: receiver operating characteristic
RR: relative risk
SaO_2_: arterial oxygen saturation
ScvO_2_: central venous oxygen saturation
SBP: systolic blood pressure
ScvO_2_: central venous oxygen saturation
SOFA: sequential organ failure assessment score
VO_2_: oxygen consumption
