# Supplementary material for: Clinical classification of tissue perfusion based on the central venous oxygen saturation and the peripheral perfusion index
Source: Crit Care. 2015 Sep 14;19(1):330. doi: 10.1186/s13054-015-1057-8 (PMC4568576; doi:10.1186/s13054-015-1057-8)
Supplement: Additional file 1: — Results: difference between the patients with peripheral perfusion index (PI) ≤0.6 and with PI >0.6 at 8 h (T8) after resuscitation. (DOCX 19 kb) [file 13054_2015_1057_MOESM1_ESM.docx]

**Clinical classification of tissue perfusion based on the central venous oxygen saturation and the peripheral perfusion index**

Huai-wu He, Yun Long, Da-wei Liu, Xiao-ting Wang, Xiang Zhou

**Result**

**Difference between the patients with PI<0.6 and with PI>0.6 at T8 after resuscitation**

| Variables  at T8 | Pts with PI>0.6  n=167 | Pts with PI<0.6  n=35 | | P-value |
| --- | --- | --- | --- | --- |
| PI  MAP  Heart rate(bpm) | 2.53±1.41  86±12  89±20 | 0.22±0.02  86±17  109±27 | <0.0001 **^a^**  0.852  <0.0001 **^a^** | |
| CVP  Temperature (°C)  P(v-a)CO_2_  ScvO_2_  Lactate | 9±3  37.4±0.8  5±3  78±9  1.6±1.3 | 10±4  37±1  8±5  74±11  3.9±4.3 | 0.128  0.055  0.007 **^a^**  0.120  0.0001 **^a^** | |

**^a^** P <0.05 for PI<0.6 pts. vs. PI>0.6 pts. Data is presented as mean ± SD MAP mean arterial pressure, CVP Central venous pressure (mmHg), MAP Mean arterial pressure (mmHg), ScvO_2_ Central venous oxygen saturation(%), P(v-a) CO_2_ veno-arterial CO_2_ tension difference(mmHg), Lactate (mmol/L), PI peripheral perfusion index.

;
