# Supplementary material for: Clinical classification of tissue perfusion based on the central venous oxygen saturation and the peripheral perfusion index
Source: Crit Care. 2015 Sep 14;19(1):330. doi: 10.1186/s13054-015-1057-8 (PMC4568576; doi:10.1186/s13054-015-1057-8)
Supplement: Additional file 2: — Results: related variables of each subset of patients based on normalized central venous oxygen saturation (ScvO 2 ) (70 %) and critical peripheral perfusion index (PI) (0.6) at 8 h (T8). (DOCX 20 kb) [file 13054_2015_1057_MOESM2_ESM.docx]

**Clinical classification of tissue perfusion based on the central venous oxygen saturation and the peripheral perfusion index**

Huai-wu He, Yun Long, Da-wei Liu, Xiao-ting Wang, Xiang Zhou

**Result**

Related Variables of Each Subset of Patients Based on Normalized ScvO_2_ (70%) and PI (0.6) at T8

|  | **Group1** | **Group2** | **Group3** | | **Group4** |
| --- | --- | --- | --- | --- | --- |
|  | PI<0.6+ScvO_2_ <70%  N=10 | PI<0.6andScvO_2_≥70%  N=25 | PI>0.6andScvO_2_<70%  N=25 | | PI>0.6andScvO_2_≥70%  N=142 |
| CVP |  |  |  | |  |
| T0 | 10±3 | 9±4 | 9±3 | | 9±3 |
| T8 | 10±4 | 9±4 | 8±3**b** | | 9±3 |
| MAP |  |  |  | |  |
| T0 | 91±18**b** | 94±21 | 92±16 | | 91±17 |
| T8 | 86±13 | 86±15 | 85±12 | | 87±12 |
| ScvO_2_ |  |  |  | |  |
| T0 | 68±11**c,** | 71±12 | 67±16**c** | | 76±10 |
| T8 | 60±10**a,c** | 80±4**b,c** | 63±7 **c** | | 80±6 |
| P(v-a) CO_2_ |  |  |  | |  |
| T0 | 8±6 | 8±4**c** | 8±6 | | 6±3 |
| T8 | 11±5**a,b,c** | 7±5 | | 7±4**c** | 5±3 |
| Lactate( mmol/L) |  |  | |  |  |
| T0 | 3.3±2 | 3.2±3 | | 2.1±1.6 | 2.7±2.1 |
| T8 | 6.4±6**a,b,c** | 3±3**b,c** | | 1.6±1.5 | 1.6±1.3 |
| PI |  |  | |  |  |
| T0 | 0.81±0.38**a** | 0.55±0.61**b,c** | | 1.6±1.2 | 1.5±1.2 |
| T8 | 0.3±0.17**b,c** | 0.36±0.18**b,c** | | 2.5±1.7 | 2.5±1.4 |

CVP Central venous pressure (mmHg), MAP Mean arterial pressure (mmHg), ScvO_2_,central venous oxygen saturation, P(v-a) CO_2_ veno-arterial CO_2_ tension difference, PI peripheral perfusion index. ap <0.05 for vs. group2; bp <0.05 for vs. group3; cp <0.05 for vs.group4.
