# Supplementary material for: Clinical classification of tissue perfusion based on the central venous oxygen saturation and the peripheral perfusion index
Source: Crit Care. 2015 Sep 14;19(1):330. doi: 10.1186/s13054-015-1057-8 (PMC4568576; doi:10.1186/s13054-015-1057-8)
Supplement: Additional file 3: — Results: relationship between peripheral perfusion index (PI) and blood gas metabolic variables on repeated measurements during resuscitation. (DOCX 18 kb) [file 13054_2015_1057_MOESM3_ESM.docx]

**Clinical classification of tissue perfusion based on the central venous oxygen saturation and the peripheral perfusion index**

Huai-wu He, Yun Long, Da-wei Liu, Xiao-ting Wang, Xiang Zhou

**Result**

**Relationship between PI and blood gas metabolic variables on repeated measurements during resuscitation.**

|  | All data of PI, n=404 |  | Data of PI<1.4 , n=204 |  | Data of PI>1.4, n=200 |
| --- | --- | --- | --- | --- | --- |
| Variables | r p-value |  | r p-value |  | r p-value |
| ScvO_2_ | 0.190 <0.0001**^*^** |  | 0.193 0.006**^*^** |  | 0.137 0.053 |
| P(v-a) CO_2_ | -0.152 <0.0001**^*^** |  | -0.240 0.001**^*^** |  | -0.061 0.392 |
| Lactate | -0.261 <0.0001**^*^** |  | -0.286 <0.0001**^*^** |  | -0.120 0.089 |

ScvO_2_, central venous oxygen saturation (%); P(v-a) CO_2_ ,venoarterial CO_2_ tension difference(mmHg);

PI , peripheral perfusion index measured by pulse oximetry; **^*^***P* <0.05.
